# The Role of Inflammation and Inflammatory Mediators in the Development, Progression, Metastasis, and Chemoresistance of Epithelial Ovarian Cancer

**DOI:** 10.3390/cancers10080251

**Published:** 2018-07-30

**Authors:** Sudha S. Savant, Shruthi Sriramkumar, Heather M. O’Hagan

**Affiliations:** 1 Medical Sciences, Indiana University School of Medicine, Bloomington, IN 47405, USA; ssavant@iu.edu; 2 Cell, Molecular and Cancer Biology Graduate Program, Indiana University, Bloomington, IN 47405, USA; ssriramk@iu.edu; 3 Indiana University Melvin and Bren Simon Cancer Center, Indianapolis, IN 46202, USA

**Keywords:** inflammation, epithelial ovarian cancer, cytokines, reactive oxygen species, growth factors

## Abstract

Inflammation plays a role in the initiation and development of many types of cancers, including epithelial ovarian cancer (EOC) and high grade serous ovarian cancer (HGSC), a type of EOC. There are connections between EOC and both peritoneal and ovulation-induced inflammation. Additionally, EOCs have an inflammatory component that contributes to their progression. At sites of inflammation, epithelial cells are exposed to increased levels of inflammatory mediators such as reactive oxygen species, cytokines, prostaglandins, and growth factors that contribute to increased cell division, and genetic and epigenetic changes. These exposure-induced changes promote excessive cell proliferation, increased survival, malignant transformation, and cancer development. Furthermore, the pro-inflammatory tumor microenvironment environment (TME) contributes to EOC metastasis and chemoresistance. In this review we will discuss the roles inflammation and inflammatory mediators play in the development, progression, metastasis, and chemoresistance of EOC.

## 1. Inflammation and EOC

Inflammation is part of the immune response that protects against foreign pathogens and aids in healing. Inflammation is elicited in response to cellular damage either by infection, exposure to foreign particles (pollutants or irritants), or an increase in cellular stress [1]. The ultimate goal of the inflammatory response is to restore tissue homeostasis, either by destruction or healing of the damaged tissue. The acute or immediate inflammatory response involves modification of the vasculature surrounding the site of stress or damage to increase blood flow. This alteration is then followed by activation of innate immune cells already present in the tissue, including macrophages, dendritic cells (DC), and mast cells, and an increase in infiltration of additional innate immune cells into the affected tissue. At sites of inflammation there are high levels of reactive oxygen species (ROS), cytokines, chemokines, and growth factors that are produced by the immune cells and other cells in the tissue. Acute inflammation is essential for tissue homeostasis and to protect against normal exposure to pathogens. However, in certain cases the body is unable to resolve this response or is subjected to repeated stimulation resulting in chronic inflammation.

Ovarian cancer (OC) is the fifth leading cause of cancer-related deaths in women in the United States [2] and can originate in the germ cells, sex-cord stroma, the fallopian tube (FT), or ovary epithelium. Epithelial ovarian cancer (EOC) which originates from the ovary or fallopian tube epithelium, accounts for 85–90% of all OCs. Chronic inflammation is an important risk factor associated for EOC and high grade serous ovarian cancer (HGSC), the most malignant subtype of EOC. Chronic inflammation results in activation of signaling pathways, transcription factors, and the innate and adaptive immune responses [3,4]. In this review we primarily focus on inflammation as a risk factor for invasive EOC, but have also included supportive evidence from other OC subtypes, studies that do not define the subtype of OC, and other tumor types as indicated.

### 1.1. Signaling Pathways and Transcription Factors

Several signaling pathways and transcription factors involved in the inflammatory response also play critical roles in EOC. Here we briefly introduce relevant pathways that will be linked to OC formation in later sections. Cytokines produced during inflammation bind to and activate toll like receptors (TLRs) on cell surfaces, which results in activation of the signaling pathways involving mitogen-activated protein kinases (MAPKs) p38 and JNK (c-Jun N-terminal kinase) and transcription factors including nuclear factor kappa-light-chain-enhancer of activated B cells (NF-κB) and the signal transducer and activator of transcription (STATs). The MAPK pathway regulates cellular processes like proliferation, differentiation, growth, migration, and cell death by upregulating the expression of transcription factors like AP-1, c-Jun, FOS and by activating NF-κB and STATs, that either by themselves or along with AP-1 or c-Jun regulate expression of pro-survival and pro-growth genes. NF-κB and AP-1 also regulate production of cytokines like IL-6 [5,6,7].

During inflammation these transcription factors play an important role to maintain tissue homeostasis. However, in case of chronic inflammation, the signaling pathways are continuously stimulated, which can contribute to tumorigenesis.

### 1.2. Innate Immune Response

Inflammation activates the innate immune response, which signals macrophages and DCs to secrete chemoattractants like Interleukin-8 (IL-8), monocyte chemotactic protein-1 (MCP-1), and various other inflammatory mediators. These chemoattractants in turn result in recruitment of neutrophils, lymphocytes, and natural killer (NK) cells to the site of damage. All of these cells then secrete cytokines like IL-1, IL-3, IL-6, IL-8, tumor necrosis factor alpha (TNF-α), interferon (IFN) α, and colony-stimulating factors (CSF) like granulocyte macrophage CSF (GM-CSF). The cytokines bind to transmembrane receptors on the cell surfaces of other cells to activate transcription factors that regulate gene expression downstream of the cytokine activated pathway. This creates a pro-inflammatory environment resulting in recruitment of other immune cells, migration of endothelial cells, and proliferation of fibroblasts. Activation of macrophages and NK cells results in the production of high levels of ROS and reactive nitrogen species (RNS), which are used by these cells to kill foreign pathogens, but also end up damaging neighboring normal cells [8]. The lymphocytes also secrete growth factors like platelet derived growth factor (PDGF), transforming growth factor beta (TGF-β), and fibroblast growth factor (FGF), which facilitate wound healing. Overall the acute immune response is a rapid response that typically only lasts a few days. It results in removal of the pathogen, release of proteolytic enzymes to destroy damaged tissue, or stimulation of the proliferation of fibroblasts and epithelial cells to repair the tissue [1].

### 1.3. Adaptive Immune Response

If the infection is not resolved by the innate immune response, the adaptive immune response is activated, which is less inflammatory in nature. The adaptive immune response also provides longstanding protection against specific pathogens and/or antigens. B cells and T cells are the effector cells of the adaptive immune system that are derived from lymphocytes when they are presented with specific antigens by the antigen presenting cells (APC). T cells respond to the APCs by producing IL-2, which induces expression of transcription factors that facilitate T cells to differentiate into T regulatory (Tregs) and T effector (Teff) cells. There are two major classes of T effector cells; CD8^+^ cytotoxic T cells and CD4^+^ T helper (Th) cells. Th cells are further differentiated into Th1, Th2, or Th17 depending on the ILs secreted and the transcription factors expressed. IFN-y activates STAT1 to induce formation of Th1 and IL-6, and TGF-β can induce Th17 cell formation. Th1 and Th17 secrete ILs and activate macrophages and B cells to create a pro-inflammatory microenvironment (ME) that can be protumorigenic depending on the context. Tregs are immunosuppressive cells that turn off the immune response [1,9,10].

## 2. Inflammation as a Risk Factor for EOC

Amongst other factors such as hereditary, environmental, and lifestyle, inflammation emerges as an important risk factor for EOC. EOC arises either in the epithelial layer surrounding the ovary or in the epithelium of the distal FT, which could then spread to the ovary. A significant portion of HGSC is thought to originate in the FT, in part because removal of the FT significantly reduces OC risk [11]. Interestingly, while surgical specimens from mutation carriers rarely had premalignant ovarian epithelial changes, early lesions called serous tubal intraepithelial carcinomas (STICs) were found in the FTs of 5–10% of the patients. Copy number and mutational analysis suggest that STICs shed cells with metastatic potential that then colonize the ovary to form HGSC. STICs are mostly found in the fimbriae, the distal end of the FT that shares a ME with the ovary. During a woman’s lifetime, the repeated secretion of ROS, cytokines, and other growth factors by the ovaries and immune cells creates a chronic inflammatory ME in the peritoneum that in turn potentiates the initiation of normal cells to malignant ones in the FT and the ovary, supports tumor progression, metastasis, and development of resistance to chemotherapy.

During ovulation, infection and other causes of inflammation ovary and FT tissue is damaged and undergoes repair. We will briefly discuss how each of these processes evoke or involve an inflammatory response that can persist, leading to a cytokine and growth factor rich environment in the peritoneum and contribute to EOC.

### 2.1. Ovulation

The process of ovulation itself is comparable to that of inflammation as described in the early 20th century. The development of the follicle to its rupture and release of the egg results in recruitment of activated immune cells to the ovary and production of enormous amounts of chemokines, cytokines, and growth factors. Ovulation is initiated by a surge of Luteinizing hormones (LH) that results in increased blood flow to the ovarian follicles. Before release of the egg, the surge of LH hormone recruits neutrophils and macrophages to the graafian follicles [12,13,14]. Macrophages in the theca have been shown to support growth of follicles [15]. During ovulation macrophages secrete growth factors like hepatocyte growth factor (HGF), TGF-β, and epidermal growth factor (EGF), which stimulate cellular proliferation and follicle growth. Simultaneously the macrophages also secrete ROS, TNF-α, and IL1β, which stimulate local apoptosis resulting in rupture of the follicle, which bathes the ovarian surface and fimbriae with follicular fluid. Exposure of FT cells to follicular fluid results in altered expression of genes associated with inflammation, including increased expression of IL8 and cyclooxygenase-2 (COX-2) [16]. Quiescent fibroblasts are present in the thecal layer surrounding the follicles. Exposure to growth factors stimulates their proliferation and they then secrete prostaglandins, collagenases, and plasminogen activator. In the corpus luteum, after the follicle is released, the macrophages secrete prostaglandins, ROS, and TNF-α, which stimulate apoptosis of the corpus luteum cells. Therefore, ovulation results in the cyclic exposure of FT and ovarian epithelial cells to high levels of ROS, cytokines, and growth factors [17] Although the other causes of inflammation discussed below are important and result in increased overall risk for EOC, the process of ovulation itself occurs often in the lifetime of the majority of women and may be the most important inflammation-related risk factor for EOC. This hypothesis is corroborated by the laying hen model, which is commonly used to study ovarian cancer [18]. In this model, hens develop spontaneous EOC, likely due to their high ovulation rate, thus linking ovulation directly as an increased risk factor for EOC. Delayed onset of menarche and early onset of menopause have been shown to be inversely related to the risk of OC, likely due to the reduction in number of ovulation cycles in a woman’s lifetime [19,20]. Further, ovulation has also been connected to EOC because contraceptive pills, pregnancy, and breastfeeding reduce the risk of OC. These factors reduce, halt, or delay overall ovulation cycles, respectively, which in turn reduces overall exposure to inflammation of the ovary and FT. The associations of parity and oral contraceptive use with invasive EOC were recently confirmed in a large, prospective study using the European Prospective Investigation into Cancer and Nutrition (EPIC) cohort that found only limited heterogeneity in the risk between reproductive factors and EOC subtypes [21]. Hysterectomy, tube ligation, and removal of ovaries are also protective against development of OC [22,23].

### 2.2. Infection

Pelvic inflammatory disorder (PID) is the infection of the female reproductive organs like cervix, uterus, FTs, and ovaries. It is a significant risk factor for OC and is caused by various bacteria and virus such as *Chlamydia trachomatis*, *Mycoplasma genitalium*, *Neisseria gonorrhoeae*, human papilloma virus, and cytomegalovirus [24,25]. Infection by these microbes results in DNA damage and production of ROS and induces a pro-inflammatory response, which involves secretion of cytokines and migration of immune cells [24]. PID is generally resolved with antibiotics within 48–72 hours of detection. However, repeated infection and unresolved inflammation can lead to chronic inflammation that is a risk factor for EOC.

### 2.3. Other Sources of Inflammation

The other causes of inflammation in the ovaries and/or FTs are endometriosis, obesity, Polycystic Ovarian Syndrome (PCOS), and talc exposure. Endometriosis is defined as presence of stroma and endometrial gland tissues in the pelvic peritoneum, rectovaginal septum, and ovaries [26]. Retrograde menstruation is the most commonly accepted theory for endometriosis. Retrograde menstruation results in aberrant accumulation of red blood cells (RBCs) and tissue, which can trigger an inflammatory response, activating the macrophages in the peritoneal cavity [27,28]. The macrophages lyse the RBCs, resulting in an increase in iron accumulation in the endometric implants and peritoneal fluid. The accumulated iron can catalyze formation of free radicals like RNS and ROS in the peritoneum and results in increased oxidative stress (OS). OS can activate NF-κB, in macrophages resulting in secretion of growth factors, cytokines, and IFNs. Around one third of women are affected by mild endometriosis, which resolves on its own over time. For the remaining cases, endometriosis results in chronic pain and inflammation, which can be resolved by excision of affected tissue or the outgrowth. However, in 45% of these cases, the endometriosis reoccurs resulting in repeated bouts of chronic inflammation [29,30].

Obese women have higher risks of EOC and HGSC and pro-inflammatory cytokines are associated with higher body mass index (BMI) levels. Adipose tissues secrete the cytokines TNF-α, IL-6, IL-8, and MCP-1, which can induce an inflammatory reaction in the peritoneum [31]. Continuous secretion of these cytokines leads to a state of chronic inflammation, which includes activation of macrophages and recruitment of NK cells and results in high levels of OS. Once the tumor has been initiated, the continuous secretion of cytokines by adipose tissue or omentum can facilitate migration of cancer cells to the omentum, promoting metastasis of the tumor into the peritoneum [30]. High levels (>10 mg/L) of C-reactive protein (CRP), a marker of global inflammation, are associated with an increased risk of EOC [32,33]. IL-6 itself is not a risk factor for EOC but in obese women IL-6 and CRP may be associated with increased EOC risk [33].

PCOS also contributes to inflammation in women and may increase risk of EOC [34]. PCOS is a hormonal disorder occurring in reproductive aged women during which ovaries may develop numerous small collections of fluid and fail to release eggs properly. Obesity, hyperandrogenism, and increased insulin resistance further characterize PCOS. Increased C-Reactive protein (CRP) and MCP-1 levels, indicative of low-level chronic inflammation, are elevated in women with PCOS [35,36,37,38]. Simultaneously chemokines like IL-18, IL-6, and TNF-α are also increased in circulation in women with PCOS [39,40,41,42]. The increase in inflammatory mediators correlates positively with BMI, suggesting that increased obesity in women with PCOS may be the source of inflammation. Increased DNA damage and OS is observed in women with PCOS, which may also increase risk for EOC [43]. Evidence linking PCOS directly to EOC is limited due to small study sizes, PCOS being associated with other EOC risk factors such as obesity, and PCOS possibly being only associated with one subtype of EOC, borderline serous [44].

Talc is a silicate mineral and exposure to it can cause inflammation of the ovaries and poses a risk hazard for development of EOC [45]. It has been proposed that talc from talcum powder used for dusting and from condoms and vaginal diaphragms can migrate up to the ovaries via retrograde flow of fluids and mucous and get lodged in the ovaries. Tubal ligation, which is protective for EOC, is thought to block the transport off talc from the lower genital tract. Talc behaves as a foreign particle, triggering an inflammatory response [46,47]. The talc attracts macrophages, which try to phagocytose it. The macrophages then send chemotactic signals to other immune response mediators and initiate a wound healing process. Since talc is not degradable by the body, it inhibits the wound healing process, resulting in chronic inflammation.

### 2.4. NSAIDS and Reduced Risk of EOC

Further connecting inflammation to EOC are several studies that demonstrate that intake of non-steroidal anti-inflammatory drugs (NSAIDs), specifically of aspirin, correlates inversely with risk of OC and endometrial cancer [48,49,50,51,52]. In vitro studies with OC cell lines and NSAIDS show that NSAIDs and COX-2 inhibitors facilitate apoptosis, however this effect is not dependent on COX-2 and may be due to upregulation of p21, a protein important for cell cycle arrest [53]. Another study by Arango et al., demonstrates that acetylsalicylic acid or aspirin resulted in increased apoptosis via downregulation of Bcl2 in an endometrial cancer cell line [54]. A third study has shown that a selective COX-2 inhibitor, JTE-522, can inhibit proliferation and increase apoptosis of endometrial cancer cells by increasing levels of p53 and p21 and decreasing phosphorylation of retinoblastoma (Rb) protein, which results in its activation; all of which results in cell cycle arrest [55,56]. Simultaneously, there was an increase in caspase-3 activity, which is indicative of increased apoptosis. Another mechanism by which aspirin could facilitate its chemopreventive nature is by inhibiting oxidative induced DNA damage [57]. COX-1 is also expressed in normal ovaries of the laying hen, with expression increasing in post-ovulatory follicles suggesting its importance for or a role in ovulation. With the onset of OC, COX-1 expression is increased [58] and COX-1 inhibition and NSAIDs have shown to decrease proliferation of ascites in the laying hen OC model [59]. Further, when 0.1% aspirin was included in their diet for one year, although the onset of OC was not different, the progression of cancer was slower when compared to hens fed with regular diet [60].

**Figure 1:**
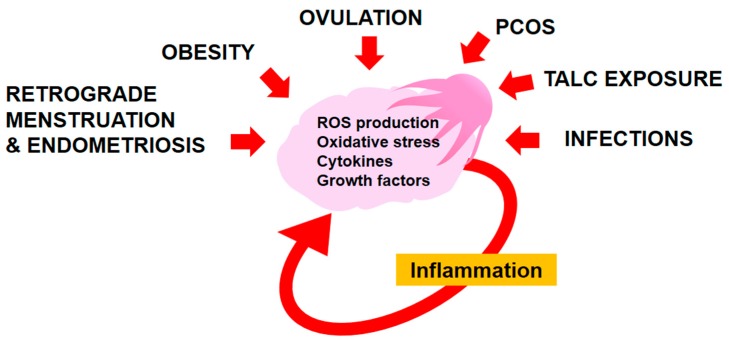
Sources of inflammation in the ovary and fimbriae. Ovulation, retrograde menstruation, endometriosis, infections, exposure to talc, Polycystic Ovarian Syndrome (PCOS), and obesity result in exposure of the ovary and fimbriae to reactive oxygen species (ROS), oxidative stress, cytokines, and growth factors, generating an inflammatory response that leads to additional production of ROS and cytokines in the ovary. Unresolved, chronic inflammation is a critical risk factor for tumor initiation.

As discussed, inflammation results in secretion of ROS, growth factors, cytokines, and chemokines into the shared environment surrounding the ovary and distal FT. Exposure of normal tissue to these inflammatory mediators results in activation of downstream signaling that can promote the transformation of normal cells or survival of already transformed cells. Once EOC has already formed further exposure of cancer cells to these inflammatory mediators also results in activation of downstream signaling within the cancer cell and in the surrounding tissue, creating an inflammatory environment that can further promote EOC (Figure 1). We will discuss in more detail how key inflammatory mediators contribute to EOC initiation, progression, metastasis, and chemoresistance.

## 3. Inflammation and EOC Initiation and Progression

Tumorigenesis is a multistep process that requires cells to gain the ability to evade apoptosis and antigrowth signals, proliferate independently of stimuli, develop a support system (angiogenesis), and have the capacity to invade and metastasize. Tumorigenesis is initiated by the transformation of a normal cell to a malignant one. The deregulation of the above mentioned processes in the malignant cell could potentiate its progression to cancer.

**Figure 2:**
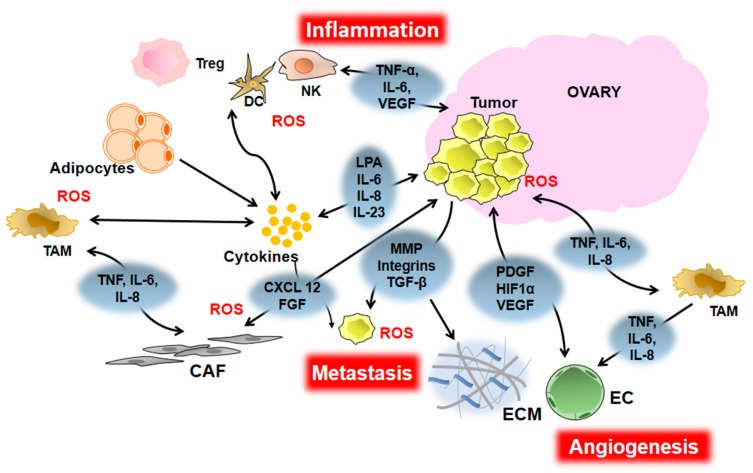
Inflammatory mediators contributing to EOC progression, metastasis, and angiogenesis. EOC cells produce ROS, chemokines, cytokines, and growth factors that can: (1) Lead to recruitment of immune cells like Dentric cells (DC), Natural killer cells (NK), Tumor associated macrophages (TAMs), and T-regulatory (Treg) cells into the TME, which generate additional cytokines, ROS, and growth factors, resulting in chronic inflammation. (2) Stimulate the tumor cells themselves, the TAMs, and the surrounding fibroblasts (also known as cancer associated fibroblasts or CAFs) to proliferate and secrete growth factors like TGF-β and FGF that stimulate production of integrins and Matrix Metalloproteins (MMPs), resulting in migration of the tumor cell via degradation of the extra cellular matrix (ECM). (3) Stimulate endothelial cells (EC) to produce growth factors like PDGF and EGF and factors like VEGF that stimulate angiogenesis. The double arrows indicate that the cells are a source of the factor as well as stimulated by it.

One mechanism of cancer initiation is genomic instability due to DNA damage [61] and EOCs exhibit a high number of chromosomal aberrations and genomic instability [62]. The most common gene mutations in HGSCs include *BRCA*, *TP53*, and genes in involved in mismatch repair and the DNA damage response [63]. A pro-inflammatory ME can also contribute to genetic instability and therefore play a role in EOC cancer initiation. A pro-inflammatory ME, which is continuously supplemented by ROS, cytokines, and growth factors, can cause DNA damage in epithelial ovarian and FT cells, switch on antiapoptotic pathways, and initiate transformation of normal cells. When cells transformed either by oncogenic alterations or by exposure to inflammation are in a pro-inflammatory ME they are able to turn on pro-survival signaling pathways rather than the senescence pathways that are normally induced by oncogene expression in normal cells. For example, disruption of the RAS pathway results in activated NF-κB signaling and upregulation of its downstream targets including cytokines like IL-1β, IL-6, and IL-8. These cytokines are upregulated in EOC patients and their increased levels correlate with decreased survival [64,65,66,67,68,69,70,71]. The inflammatory mediators like cytokines, chemokines, growth factors, and prostaglandins secreted by the transformed epithelial cells further promote a pro-inflammatory environment, which can reprogram the surrounding cells to form the TME. The TME is mainly composed of endothelial cells, cancer associated fibroblasts (CAFs), adipocytes, tumor associated macrophages (TAMs), regulatory T-cells, pericytes, infiltrated immune cells such as neutrophils, lymphocytes, and various other cells that further secrete growth factors and cytokines which potentiate tumor progression (Figure 2, Table 1). Furthermore, OC-initiating cells (OCICs) have been identified in tumors and ascites that exhibit stem cell like properties and are capable of forming tumors [65,66,72]. Cytokines can promote self-renewal of CD133^+^ OCICs to potentiate tumor progression [73].

The innate immune response can prevent tumorigenesis by recognizing and eliminating transformed cells. However, chronic inflammation can contribute to the ability of premalignant cells to evade apoptosis, escape the immune surveillance, and continue to grow, resulting in tumor formation. As mentioned, EOC can originate from either distal FT or ovarian epithelial cells. Since both the ovary and fimbria are exposed to the same ME, exposures reviewed here are relevant to initiation in either tissue. [74]. In this section we will review the role of OS and some specific pro-inflammatory mediators and signaling pathways in the initiation and progression of EOC.

### 3.1. ROS and Oxidative Stress

ROS plays an important role in the normal female reproductive cycle, from affecting maturation of the oocyte to ovulation, apoptosis of cells in corpus luteum, and embryo development [75]. Ovulation results in increased levels of DNA damage in the FT epithelium that is likely a result of the ROS generated during ovulation or the ovulation-associated increase in numbers of infiltrating macrophages in the FT [17]. Additionally, during infection and inflammatory responses immune and damaged cells produce ROS resulting in continuous exposure of the ovaries, FTs, and peritoneal cavity to ROS [76,77,78]. ROS exposure could potentially lead to epithelial cells in the ovary and FT undergoing transformative changes, as has been demonstrated for ovarian surface epithelium cells grown in 3D culture [79]. Elevated ROS and RNS levels beyond the level that cells can neutralize results in OS. Increased OS results in DNA damage, activation of signaling cascades, and epigenetic alterations.

DNA damage in a cell results in stimulation of DNA damage repair pathways. These repair pathways can be inactivated or be erroneous, which results in increased genotoxic stress and mutated DNA. Secretory tubal epithelial cells in the FT, a cell of origin for HGSC, are particularly susceptible to genotoxic injury with persistent DNA damage that could lead to mutation and STIC formation [80]. Mutations in tumor oncogenes and suppressors result in overexpression, constitutive activation of the protein, loss of expression, or expression of nonfunctional proteins, resulting in a transformed cell. Follicular fluid may have transformative properties as it has been demonstrated that bathing fimbriae with follicular fluid containing high levels of ROS results in increased levels of DNA damage. Bathing fimbriae that have loss of p53 and Rb with this follicular fluid results in evasion of apoptosis and cells with persistent DNA damage [81].

ROS can activate pro-survival intracellular tyrosine phosphorylation signaling cascades, mainly regulated by the MAPKs and redox sensitive kinases. Activation of c-Jun, JNK, ERK (extracellular signal-regulated kinase), and p38-MAPK signaling cascades results in upregulation of cell cycle proteins that enhances proliferation. Activation of JNK can also activate NF-κB, which can suppress apoptosis. The MAPK pathway inhibits apoptosis and regulates differentiation. When activated in transformed cells these pathways are important for tumor initiation. ROS affects redox sensitive factors like thioreoxin, which is also found elevated in OC cell lines [82]. Thioredoxin is involved in redox regulation of transcription factors such as NF-κB, NRF2, forkhead box class O (FOXO) proteins, reducing factor-1 (ref-1), and hypoxia inducible factor (HIF-1α), thereby increasing their binding to the DNA. Most of these transcription factors promote tumor growth and progression by regulating expression of genes that affect cell survival and growth [83,84]. For example, FOXO, NRF2, and ref-1 transcription factors upregulate transcription of anti-oxidant proteins that scavenge free radicals and allow survival of damaged or transformed cells [85]. HIF-1α upregulates the antiapoptotic factor, bcl-2 as well as vascular endothelial growth factor (VEGF), a factor important for angiogenesis.

OS has also been shown to facilitate epigenetic mechanisms in many cancers, including EOC [86]. Innate immune-mediated inflammation drives epigenetic silencing of tumor suppressor genes (TSGs) [87]. At sites of inflammation high levels of OS result in oxidative DNA damage that is recognized by the mismatch repair proteins mutS homolog MSH2 and MSH6. MSH2 and MSH6 then recruit epigenetic silencing proteins, including DNA methyltransferase 1 (DNMT1) to the sites of damage [88]. In an in vivo model of inflammation-driven colon tumorigenesis this early recruitment to sites of oxidative DNA damage results in permanent methylation of TSGs in tumors that form at the sites of inflammation [89]. While such a mechanism has not been directly proven in EOC models, Sapoznik et al. have demonstrated that exposure to follicular fluid or inflammation can induce Activation-Induced Cytidine Deaminase (AIDS) in fallopian tube epithelial cells, which results in epigenetic and genetic changes, increase in DNA damage and genotoxic stress and may be a contributing factor to EOC [90].

### 3.2. TNF-α

The cytokine TNF-α plays an important role in the process of ovulation and in removal of damaged corpus luteum. TNF-α ligand and its receptors, TNFRI and TNFRII are upregulated in ovarian tumors compared to normal ovarian tissue and high levels of TNF-α are found in ascites from OC patients [91,92,93]. OC cells have also been shown to secrete high levels of TNF-α as compared to normal ovarian epithelial cells resulting in autocrine upregulation of TNF-α mRNA and in expression of other pro-inflammatory cytokines, chemokines, and angiogenic factors like IL-6, M-CSF, CXCL2, CCL2, and VEGF [93,94]. Kellie et al. have shown using mouse models that TNF-α stimulates IL-17 production via TNFRI resulting in myeloid cell recruitment to the ovarian TME and increased tumor growth [95]. TNF-α, also upregulates AIDS transcript levels which can contribute to genotoxic stress [90].

### 3.3. IL-6

The cytokine IL-6 has been associated with poor survival in OC and is emerging as a potential therapeutic target for EOC [67,68,96,97]. IL-6 is normally produced by ovarian epithelial and OC cells. Macrophage migration inhibitory factor (MIF), EGF, and Transglutaminase secreted by OC cells can stimulate IL-6 production via activation of NF-κB [98,99,100]. IL-6 increases proliferation of OC cells by facilitating their exit from G1 into S phase of the cell cycle and by activation of the MAPK-ERK-Akt (protein kinase B) growth promoting signaling pathway [101]. ERK activation can promote formation of ascites by increasing the migration of tumor cells [70]. IL-6 production by M2 macrophages present in ascites in later stages of EOC can also stimulate cancer cell proliferation via STAT3 activation [102]. High levels of IL-6 can result in immune suppression by downregulation of IL-2, which stimulates Teff cell production [103]. IL-6 also stimulates production of Metallomatrix proteins (MMPs) in OC cells, which increases their invasive properties and promotes tumorigenesis [101,104].

### 3.4. IL-8

IL-8 a member of C-X-C chemokine family is present in the preovulatory follicle [105] where it may play a role in increasing leukocyte infiltration [106]. It is also elevated in ovarian cysts and in OC patients compared to healthy controls [107,108]. IL-8 has been found to be present in significantly higher levels in the ascites of patients with OC in comparison to patients with benign gynecological disorders [109]. Increased IL-8 expression has been associated with poor prognosis in OC patients [107]. Treatment of EOC cells with IL-8 results in their increased proliferation, which is accompanied by an increase in cyclins B1 and D1 and is dependent on phosphorylation of Akt and ERK [110]. Cyclins B1 and D1 are important for cell cycle progression, and an increase in their expression leads to increased cell growth. On the other hand, two independent studies have demonstrated that IL-8 inhibits EOC growth by increasing neutrophil infiltration [111,112].

### 3.5. Lyophosphotidic Acid (LPA)

LPA is a phospholipid that binds to and activates the endothelial differentiation gene (Edg) family of receptors. LPA is present in ovarian follicular fluid and it stimulates IL-6 and IL-8 production in the corpus luteum [113,114]. OC cells have been shown to produce LPA, which functions like a growth factor [115,116,117,118,119]. Plasma and ascites of OC patients have elevated levels of LPA that contribute to OC progression via upregulation of COX-2 and MMP2 [115,120,121]. LPA can bind to LPA_2_ receptor and induce expression of IL-6 and IL-8 via activation of NF-κB and AP-1 in OC cell lines [122]. It can induce ROS dependent Akt and ERK phosphorylation and inhibition of LPA can increase apoptosis of EOC cells [123]. ERK phosphorylation can induce phosphorylation of HIF-1α, which then can upregulate VEGF and promote tumorigenesis. Another group demonstrated that stimulating EOC cells with ether-linked LPA resulted in their increased proliferation and survival by increased synthesis of DNA and activation of Akt via PI3K, which contributes to tumor progression [124].

### 3.6. Prostaglandins and COX-1 and COX-2

Prostaglandins are secreted in the ovary, FT, and uterus. They are important for maturation of the oocyte and facilitate the movement of the FT so that the mature oocyte can move from the ovary to the uterus. In the uterus prostaglandins help regulate and maintain uterine blood flow. COX-1 and COX-2 are enzymes that catalyze the production of prostaglandins from arachidonic acid and are overexpressed in OC patients [22,125,126]. High COX levels positively correlate with increased cell proliferation, angiogenesis, and malignancy in ovarian tumors [126,127]. COX-1 and COX-2 are normally involved in the acute inflammatory response but can become dysregulated in chronic inflammatory or TMEs. Obermajer et al. have demonstrated that prostaglandins produced by COX-2 can stimulate production of CXCR4 and its ligand Stromal cell derived factor 1 (SDF1) CXCL12 in myeloid derived suppressor cells (MDSC), which stimulates them to migrate towards OC ascites [128]. MDSCs inhibit the proliferation and differentiation of T cells, resulting in overall immune suppression, which allows the tumor cells to escape immune surveillance and continue to grow. Genetically engineered mouse models of EOC; one harboring the *p53* and *Rb* deletion and other the *KRAS^G12D^* mutation and *Pten* deletion, demonstrate increased COX-1 levels, thus suggestive that COX-1 could be used as a potential biomarker and therapeutic target for EOC [129]. Further when COX-1 was inhibited in EOC cells, it led to reduction in prostacyclin (a type of prostaglandin) synthesis and reduced tumor growth by enhanced apoptosis [130].

## 4. Inflammation and EOC Angiogenesis

Angiogenesis is required for the growth of both primary and metastatic tumors [131]. The process of angiogenesis is a complex multi-step process reviewed previously [132]. It is regulated by a balance between pro-angiogenic and antiangiogenic factors. Hypoxic and ischemic areas are present at sites of inflammation and also in tumors mainly due to obstruction of local blood vessels, differences in pace of growth of blood vessels and growth of the tumor and/or infiltration of immune cells. Macrophages accumulate at hypoxic sites and alter their gene expression profiles in response to the hypoxic conditions. One of the important genes for angiogenesis that is upregulated by hypoxia is VEGF [133,134]. The rate-limiting step in angiogenesis is VEGF signaling in endothelial cells (ECs) [135]. VEGF functions via tyrosine kinase receptors VEGF-1 and VEGF-2 and promotes migration, survival, proliferation of ECs, and formation of new blood vessels [136,137,138]. Many of the inflammatory mediators discussed so far are also involved in promoting angiogenesis in EOC as detailed below (Figure 2, Table 1).

### 4.1. TNF-α

TNF-α creates a pro-inflammatory TME and has also been associated with promoting angiogenesis. It has been hypothesized that TNF-α induces the production of soluble factors that promote tumor angiogenesis. Culture supernatants from TNF-α expressing cells induce the growth of mouse lung endothelial cells in vitro while culture supernatants from TNF-α lacking cells do not exert the same effect [94]. In pituitary adenomas TNF-α is known to induce VEGF that in turn induces CXCL12 [139,140]. VEGF and CXCL12 synergistically induce angiogenesis in EOC [141]. Mice injected with OC cells lacking TNF-α have reduced vascular density in their tumors and reduced formation of blood vessels in the peritoneal deposits. These mice also did not have accumulation of ascetic fluid suggesting the importance of TNF-α in angiogenesis and EOC progression [94].

### 4.2. IL-6

In physiological conditions, IL-6 is involved in angiogenesis in the ovary during the development of ovarian follicles [142]. IL-6 induces the phosphorylation of STAT3 and MAPK in ovarian endothelial cells thereby enhancing their migratory ability, a key step in angiogenesis [143]. As explained before, OC cells also secrete increased amounts of IL-6. Some OC cells also secrete an alternative splice variant of IL-6Rα, the soluble form sIL-6R, which consists of only the ectodomain of the transmembrane receptor. By a process called trans-signaling, the sIL-6R-IL-6 complex initiates signaling in cells in the ME that do not express the transmembrane receptor facilitating angiogenesis [144].

### 4.3. IL-8

Several studies have clearly established the role of IL-8 in promoting angiogenesis. Hu et al., demonstrated that IL-8 plays a role in angiogenesis using a rat sponge model [145]. IL-8 was also able to induce angiogenesis in the rat cornea, which is normally avascular [146]. As explained in the previous section, there are several sources of IL-8 in ovarian TME. Overexpression of IL-8 in A2780 (non-IL-8 expressing) OC cells has been shown to increase the expression of VEGF, MMP-2, and MMP-9; while depletion of IL-8 in SKOV3 (IL-8 expressing) cells has been shown to reduce VEGF, MMP-2, and MMP-9 [110]. The process of angiogenesis involves degradation of extracellular matrix components and proliferation and migration of endothelial cells. MMPs are a family of endopeptidases that breakdown components of extracellular matrix and have been implicated in angiogenesis [147]. Because of the importance of VEGF and MMPs in angiogenesis these findings suggest that IL-8 in the ovarian TME will promote the formation of new blood vessels in EOC. Targeting IL-8 using mouse models reduces EOC growth and decreases angiogenesis [112].

### 4.4. LPA

In addition to playing a role in initiation, and progression, LPA has also been implicated in angiogenesis in OC. LPA has been shown to induce transcriptional activation of VEGF in EOC cell lines [163]. Transcriptional activation of VEGF primarily occurs through HIF-1α under oxygen limiting conditions in Hep3B hepatocellular carcinoma cells [164]. LPA mediated induction of VEGF expression has been shown to be independent of HIF-1α in EOC cell lines. Transition metal cobalt treatment also leads to stabilization of HIF1α similar to hypoxia. Combination treatment of EOC cells with cobalt and LPA additively increased VEGF production suggesting the effect of two different pathways [155]. LPA activates c-Myc and Sp-1, which induce VEGF expression through consensus binding sites in the VEGF promoter that have been implicated in HIFα independent induction of VEGF [155].

## 5. Inflammation and EOC Metastasis

Tumor metastasis is the major cause of mortality in most cancers, including EOC. Most EOC patients are diagnosed at an advanced stage when the cancer has already metastasized [165]. Dissemination of cancer cells to distant sites is a complex multi-step process called the invasion-metastasis cascade and is reviewed in detail in previous papers [166,167,168]. Briefly, some major steps in metastasis are—invasion through the basement membrane, intravasation into the lymphatics and circulation, survival of disseminating cancer cells in circulation, extravasation into surrounding tissues, colonization, and finally, formation of micro and macro metastases. However, unlike other epithelial malignancies, EOC has a different pattern of metastasis. EOC cells directly shed from the primary tumor into the peritoneal space and disseminate to organs in the peritoneal cavity. One of the prerequisites for cancer cells to metastasize is to undergo a process called epithelial to mesenchymal transition (EMT) where they lose their ability to attach to the basement membrane and acquire a mesenchymal phenotype and characteristics. Several recent evidences have indicated that the TME aids tumor cells to acquire these properties facilitating the metastatic cascade. An example of the ME promoting metastasis is the presence of STICs in the distal part of the FT, which shares its ME with ovary. Yang-Hartwich et al. have demonstrated that granulosa cells in the ovary secrete SDF-1 (stromal cell-derived factor 1) [169]. SDF-1 functions as a chemoattractant and recruits malignant FT cells to the ovary suggesting that the ovary is a primary site of metastasis, not the primary tumor site. Russo et al. demonstrated that loss of PTEN (phosphatase and tensin homolog) by the malignant FT cells and upregulation of WNT4 (wingless-related MMTV integration site 4) is crucial for initial metastasis to the ovary thereby supporting the tubal origin of EOC and the ovary as the primary site of metastasis [170]. The cells that make up the TME also secrete various inflammatory mediators, which facilitate progression and metastasis of OC cells (Figure 2, Table 1). These factors enable tumor metastasis by deregulating signal transduction pathways. Examples include the PI3-Akt and RAS-ERK pathways, which control migration and invasion through downstream effectors like Rho family GTPases, extracellular proteases, integrins, matrix associated proteins like focal adhesion kinases (FAK), and transcription factors like ETS2 and AP-1 [171,172,173]. Robinson-Smith et al. demonstrated that peritoneal inflammation correlated with dissemination of cancer cells from the ovaries in SCID mice. Augmenting the inflammatory response using thioglycolate accelerated ascites formation and metastasis while suppressing the inflammation using acetyl salicyclic acid impeded ascites formation and reduced metastasis. This inflammation-induced metastasis of OC cells was found to be primarily mediated by macrophages and not neutrophils or NK cells [174]. As explained in one of the previous sections a pro-inflammatory environment can be created in the peritoneum due to secretion of cytokines like IL-6 and TNF-α by adipose cells [31]. Omentum, the primary site of metastasis of OC, is largely composed of adipose cells. In addition to adipocytes, omentum also consists of blood and lymph vessels, immune cells, and stromal cells [175]. Adipocytes have been shown to increase migration, invasion, and proliferation of EOC cells. Upregulation of SUSD2 a secreted tumor suppressor by adipocytes by guadecitabine treatment reduced EOC migration and invasion. This finding suggests that epigenetic changes in the stromal cells in addition to EOC cells can facilitate EOC metastasis [176]. Omentum has aggregates of immune cells around the vasculature commonly referred to as milky spots [177]. Melanoma, lung carcinoma, ovarian carcinoma, and mammary carcinoma cell lines have been shown to specifically metastasize to the immune cell aggregates in the omentum when injected intraperitonealy into C57BL/6 mice [178]. These milky spots in the omentum have also been shown to facilitate metastatic colonization of the OC cells. Clark et al. have suggested that both adipocytes and milky spots have specific and important roles in metastatic colonization of OC cells [179]. These evidences imply that omentum potentially provides a good niche for the growth of ovarian cancer cells. Here we will specifically discuss how inflammatory mediators promote tumor metastasis in EOC.

### 5.1. ROS

EOC cells produce a large amount of ROS [180]. Loss of E-cadherin is one of the characteristic features of tumor cells with increased ability to migrate and invade. Wang et al. demonstrated that ROS leads to HIFα mediated activation of lysl oxidase. Lysl oxidase was shown to inversely correlate with E-cadherin expression promoting migration and invasion in EOC cells [181]. Tumor cells treated with sub-lethal doses of H_2_O_2_ failed to attach to the extracellular matrix components fibronectin and laminin and had increased metastatic colonization of lung, thereby establishing a role for ROS in tumor cell metastasis [182].

### 5.2. TNF-α

TNF-α provides a good example of how interactions between cancer and stroma aid in OC metastasis. Ascitic fluid and OCs contain a large number infiltrating macrophages in part because OCs constitutively produce M-CSF, which functions as a chemoattractant for monocytes [183]. These infiltrating monocytes produce many cytokines one of which is TNF-α [184,185]. OC cells also have elevated TNF-α expression that is regulated by DNA hypomethylation and chromatin remodeling of the TNF-α promoter. Increased TNF-α produced by OC cells and macrophages stimulates increased expression of TGF-α in stromal fibroblasts. TGF-α secreting stromal fibroblasts promote peritoneal metastasis of OC via EGF receptor signaling [148].

Furthermore, in EOC cells and clinical biopsies TNF-α expression correlates with one of the most commonly expressed cytokine receptors CXCR4. TNF-α stimulation of EOC cells enhanced their migration toward the only CXCR4 ligand, CXCL12. Stimulation of EOC cells by CXCL12 induced mRNA and protein expression of TNF-α. Therefore, a positive feedback loop has been suggested where in CXCL12 induced TNF-α potentially acts on the cancer cells and induces CXCR4 expression thereby enhancing tumor cell migration [149,150].

### 5.3. IL-6

IL-6 has also been implicated in metastasis of OC. Elevated levels of IL-6 found in serum and peritoneal fluid of EOC and OC patients have many sources [186,187,188]. Mesothelial cells in the peritoneum, TAMs, and EOC cells all secrete IL-6 [67]. M2 polarized macrophages in the ovarian TME induce proliferation and invasion of EOC cells by secretion of IL-6 [189]. Increased IL-6 present in ascites from OC patients enhanced the invasive ability of OC cells via the JAK-STAT signaling pathway. Canonically IL-6 signaling occurs by binding of the ligand to its transmembrane receptor IL-6Rα. The effect of IL-6 on invasion of OC cells correlated with their IL-6R expression [151]. Because through trans-signaling, the sIL-6R–IL-6 complex initiates signaling in cells that do not express the transmembrane receptor [144], we hypothesize that IL-6 produced by macrophages could also promote invasion of OC cells similar to the mechanism of induction of angiogenesis.

### 5.4. IL-8

Increased proliferation, anchorage independent growth, and angiogenic potential are some prerequisites for cells to metastasize. IL-8 increases the proliferation of OC cells and upregulates VEGF and MMP2 and 9 via activation of NF-κB, which results in enhanced invasive phenotype of OC cells. IL-8 has been shown to activate TAK1/NF-κB signaling via CXCR2, thereby facilitating the seeding and growth of OC cells in the peritoneal cavity during metastasis [153].

### 5.5. LPA

LPA promotes proliferation, survival, and metastasis of EOC cells by inducing the expression of c-Myc, VEGF, IL-8, MMPs and COX-2 [163,190,191,192,193]. LPA acts through its receptors LPAR1-3, which are members of G-protein coupled receptor superfamily. Invasive EOC cells have significantly higher expression of LPAR1 in comparison to non-invasive cell lines and LPA induces EOC cell invasion specifically through LPAR1 and not through LPAR2 or LPAR3 [194]. It can also induce secretion of urokinase in EOC cells, which has been shown to play a role in metastasis and its high levels correlate with advanced OC and poor survival in patients. LPA has been shown to increase promoter activity, mRNA levels, protein levels, and enzyme activity of Urokinase plasminogen activator (uPA) possibly via the edg-4 LPA receptor [156]. uPA is involved in converting plasminogen to plasmin, which facilitates the degradation of basement membrane and extracellular membrane proteins like fibronection aiding in metastasis [157].

### 5.6. TGF-β

TGF-β initiates signaling by dimerization of serine/threonine kinase receptors. The dimerization of receptors results in their phosphorylation, which then relays signals downstream via SMAD dependent and SMAD independent pathways. Phosphorylation by the TGF-β receptor causes R-SMADs to bind to Co-SMAD and translocate to the nucleus, where they activate transcription of genes that promote invasion, migration. Bone morphogenic proteins (BMPs) are cytokines that belong to TGF-β family and have been associated with progression of many different cancer types. Their mechanism of promoting tumor progression depends on the TME in which the cancer grows and their mode of metastatic spread [195]. Specifically, BMP-2 overexpression has been associated with poor prognosis in OC [196]. Additionally, TGF-β could potentially modify the TME to promote tumorigenesis. Veriscan (VCAN), an extracellular matrix associated protein, was upregulated by TGF-β through TGF-β receptor II (TGFBR2) and SMAD signaling making the EOC cells more aggressive. Increased VCAN expression enhanced motility and invasion of EOC cells by activating NF-κB signaling, increased expression of MMP-9, and hyaluronidase mediated motility receptor [160]. CAFs have higher expression of TGF-β receptors in comparison to normal ovarian fibroblasts and EOC cells suggesting that CAFs within the TME are more responsive to TGF-β then the other cell types [160].

## 6. Inflammation and EOC Chemoresistance

**Figure 3:**
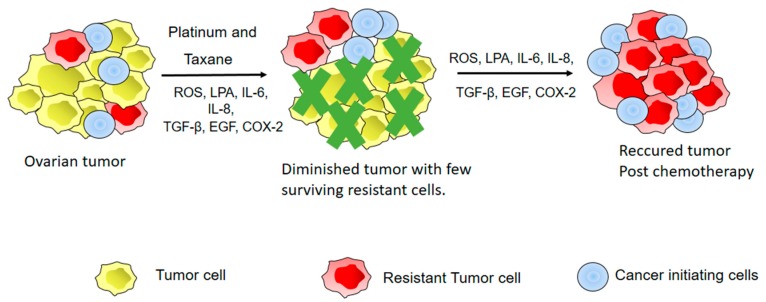
Inflammatory mediators contribute to chemoresistance of EOC. A combination of platinum and taxane drugs is currently used as chemotherapy for OC. ROS, Lyophosphotidic Acid (LPA), cytokines, and growth factors like TGF-β and EGF increase tumor cell survival by upregulating antiapoptotic genes, by stimulating stemness and proliferation of cancer initiating cells, by increasing repair of damaged DNA, or by increasing efflux of the drug. The resistant tumor cells and the cancer initiating cells can then proliferate under the influence of growth factors and cytokines resulting in a recurrent chemoresistant tumor.

The standard treatment for EOC patients is cytoreductive surgery followed by platinum/taxane-based chemotherapy [197]. The main obstacle in treatment of EOC patients is development of chemoresistance. Resistance to chemotherapy can be either intrinsic or acquired. Inherent gene expression patterns harbored by chemo-naïve tumor cells contribute to intrinsic resistance. Acquired resistance is a consequence of different alterations induced after exposure to chemotherapeutic agents [198]. Different mechanisms, including increased drug efflux, decreased uptake of the drug, inactivation of the drug, increased DNA repair, and reduced apoptotic response, have been implicated in development of platinum resistance [199]. Several recent studies have demonstrated that the TME contributes to both intrinsic and acquired resistance. One type of intrinsic drug resistance influenced by the TME is referred to as environment mediated drug resistance (EMDR). In EMDR, factors and cells present in the TME activate diverse signaling events, transiently protecting the tumor cells from undergoing apoptosis in response to chemotherapeutic agents [200,201]. Another type of drug resistance induced by cytokines, chemokines, and growth factors secreted by fibroblast cells in the tumor stroma is called soluble factor mediated drug resistance (SFM-DR). A good example of SFM-DR is IL-6 mediated drug resistance in multiple myeloma. IL-6 is important for growth of multiple myeloma cells. IL-6 activates STAT3 signaling in these cells and protects them from Fas mediated apoptosis by upregulating antiapoptotic protein Bcl-X_L_ [202]. Myeloma cells that produced IL-6 in an autocrine manner were found to be resistant to dexamethasone induced apoptosis while non-IL-6 producing cells were sensitive [203]. Cell adhesion mediated drug resistance (CAM-DR) occurs due to adhesion of tumor cells to extracellular matrix components like laminin, collagen, and fibronectin or due to fibroblasts present in the tumor stroma [204]. An example of this type of resistance is when drug sensitive myeloma cells were adhered to an extracellular matrix component fibronectin, they exhibited a reversible drug resistant phenotype which was not due reduced drug accumulation or increase in antiapoptotic proteins like Bcl-X_L_ [201]. Here we will discuss specific inflammatory mediators and their role in OC chemoresistance (Figure 3).

### 6.1. ROS

ROS are abundant in the pro-inflammatory TME. Malignant EOC tissues have been shown to have 96% higher ROS levels than normal controls [205]. OC stem like cells or OCICs are more drug resistant and responsible for relapse of chemoresistant tumors [66]. OCICs produce ROS and superoxide. This ROS induces the expression of peroxisome proliferator-activated receptor-gamma coactivator (PCG)-1α, which regulates mitochondrial biogenesis and is required for expression of detoxifying enzymes [206,207]. PCG1α increases the aldehyde dehydrogenase (ALDH) activity and expression of multidrug resistance gene (MDR1). MDR1 is an ATP dependent transporter that has been associated with efflux of platinum based drugs from OC cells contributing to platinum resistance. Scavenging ROS reduced expression of PCG1α and drug resistant related genes thereby linking ROS to development of chemoresistance [207].

### 6.2. IL-6

IL-6 in the OC TME is associated with increased chemoresistance. Wang et al. demonstrated that autocrine production of IL-6 by EOC cells makes them resistant to cisplatin and paclitaxel by causing decreased proteolytic cleavage of capase-3. Paclitaxel resistant EOC cells have increased expression of IL-6 and one of its downstream effectors STAT3 [208,209]. IL-6 producing OC cells also had increased expression of multidrug resistant genes MDR1 and GSTpi and anti-apoptotic genes Bcl-2, Bcl-xL, and XIAP, suggesting that IL6 promotes drug resistance by increasing drug efflux and reducing apoptosis [152].

### 6.3. IL-8

IL-8 blocks TRAIL-induced apoptosis and reduces caspase cleavage in EOC cell lines by decreasing the expression of death receptor (DR) 4 [210]. TRAIL is a cell death inducing ligand that belongs to the TNF superfamily and has been shown to induce apoptosis specifically in tumor cells and not in nontransformed cells [211,212]. Combination of TRAIL and the chemotherapeutic drugs—cisplatin, doxorubicin, and paclitaxel has been shown to induce apoptosis in chemoresistant EOC cell lines by causing increased caspase and PARP cleavage [154]. This finding suggests that IL8 may contribute to chemoresistance by blocking TRAIL.

### 6.4. LPA

LPA has been shown to contribute to platinum resistance by preventing cells from undergoing cisplatin-induced apoptosis without affecting their proliferation rate. The mechanism of how LPA inhibits apoptosis in EOC cells in response to cisplatin is not yet clearly understood [161].

### 6.5. TGF-β and EGF

Recurrent OC show significantly higher expression of TGF-β1 and TGF-β3 in comparison to primary tumors and normal ovary tissue [213]. Inhibition of TGF-β by the inhibitor LY2109761 sensitizes resistant SKOV3 cells to cisplatin suggesting that TGF-β contributes to the development of platinum resistance in EOC cells [162]. Cisplatin resistant A2780P cells had hypomethylation and upregulation of TGFBR2 confirming the involvement of the pathway in acquisition of platinum resistance [214]. An elevated level of EGF receptor (EGFR) has also been associated with poor prognosis in OC patients [215]. EGF has been shown to stimulate the growth of EOC cells expressing EGFR and alters their cell cycle distribution [216]. EGF similar to LPA has been shown to protect EOC cells from undergoing cisplatin induced apoptosis [161].

### 6.6. COX-2

In addition to being associated with tumor initiation and progression, COX-2 has also been associated with chemoresistance. Ferrandina et al. reported that a statistically significant higher percentage of primary OC patients unresponsive to platinum-containing chemotherapy were positive for COX-2 than responsive patients (84.6% versus 34.6%, respectively) [217]. The percentage of positive COX-2 staining per tumor area in COX-2 positive patients ranged from 15 to 45%. The results from this study suggest that COX-2 levels may influence the response of patients to different chemotherapy regimens, but the sample size of this study was small and the results need to be confirmed in a larger group of patients. Furthermore, this association needs to be corroborated biochemically [217]. In both patients groups undergoing cytoreductive surgery and explorative laparotomy, COX-2 expression was higher in nonresponders [218]. Using lung, colon, and prostate cancer models, COX-2 has been shown to induce Bcl-2 and promote tumor growth by facilitating the formation of new blood vessels [158,159]. These findings suggest that COX-2 may contribute to chemoresistance by inhibiting apoptosis and promoting angiogenesis in OC as well.

## 7. Treatment Strategies Targeting Inflammatory Mediators in EOC

As discussed, development of resistance to available chemotherapeutic drugs remains the major obstacle in management of OC patients. While several immunotherapies have been developed to improve the antitumor response of T-cells and/or modulate the immune response, here we will discuss EOC treatment strategies that specifically target the inflammatory mediators that have been reviewed above.

A monoclonal antibody directed at VEGF, bevacizumab, has been widely studied and is a promising target in EOC [219]. Bevacizumab is a recombinant humanized monoclonal antibody and has been approved by the FDA for treatment of metastatic breast, non-small cell lung, and colorectal cancer. Phase II clinical studies have shown that it is active in treatment of recurrent OC patients [220]. OCEANS trial was a randomized phase III clinical trial that evaluated the safety and efficacy of bevacizumab in combination with gemcitabine and carboplatin (GC) in comparison with GC alone in recurrent platinum sensitive ovarian, primary peritoneal, or FT cancer. This trial demonstrated that bevacizumab was able to prolong the PFS in platinum-sensitive recurrent EOC patients [221]. In addition to OCEANS, GOG218, and ICON7 have also shown that bevacizumab prolongs the PFS in OC patients confirming the promise this therapeutic target holds for management of OC [222,223].

We have discussed some mechanisms by which the pro-inflammatory cytokine TNF-α promotes OC metastasis and angiogenesis making it a good target for development of therapeutic agents. The safety profile and biological activity of a monoclonal anti-TNF-α antibody, Infliximab was assessed in a clinical study consisting of patients with advanced solid tumors, including OC. Infliximab did not have any toxic effects and was well tolerated by these patients. Reduced plasma levels of IL-6 and CCL12 in these patients was observed 24 h and 48 h after administration of Infliximab, while neutralization of TNF-α was detected after an hour indicating some biological activity [224]. This response warrants further study of Infliximab as a therapeutic agent for treatment of OC.

IL-6/STAT3 signaling has been implicated at different stages of OC progression and is a promising target although most agents are still in preclinical or early clinical trial stages. Siltuximab, an anti-IL-6 antibody, suppresses IL-6-induced STAT3 phosphorylation and nuclear translocation in OC cell lines. Siltuximab treatment also reduced the level of pro-survival proteins like Bcl-X_L_ and Survivin, which are downstream of STAT3. Siltuximab was able to sensitize paclitaxel resistant OC cell lines, but did not show the same effect in vivo [225]. sc144 is a novel small molecule inhibitor has shown significant promise in preclinical studies. sc144 binds gp130, which is a signal transducer in STAT3 signaling. It causes phosphorylation of gp130 leading to its deglycosylation. This abrogates downstream STAT3 phosphorylation and nuclear translocation inhibiting transcription of downstream genes. sc144 has increased potency in EOC cells in comparison to normal epithelial cells and slows down the growth of tumors in xenograft models of EOC [226]. A phase I clinical trial combining carboplatin, the monoclonal antibody Tocilizumab, which blocks IL-6R, and immune enhancer INF-α showed good promise. The EOC patients who received the highest dose of Tocilizumab had increased serum levels of IL-6 and sIL-6R and also showed longer median overall survival [227].

We have discussed the role of TGF-β in EOC tumor progression substantiating it as a good therapeutic target. A preclinical study of LY2109761 (TGFβRI and TGFβRII kinase inhibitor) in combination with cisplatin was conducted by Gao et al. This inhibitor significantly increased apoptosis in cisplatin resistant cells. Combining LY2109761 with cisplatin had antiproliferative effects and increased the rate of apoptosis in parental and cisplatin resistant xenograft models [162]. In triple negative breast cancer, LY2157299 a TGF-β1 receptor kinase inhibitor, prevented recurrence of tumors in xenograft models after treatment with paclitaxel [228]. Early phase clinical trials of LY2157299 in patients with advanced or metastasized pancreatic cancer have been completed. Early phase trials in triple negative metastatic breast cancer, unresectable hepatocellular carcinoma, and metastatic castration resistant prostate cancer are underway [229].

EGF has also been associated with chemoresistance in EOC. Cetuximab, a chimerized monoclonal antibody that targets EGFR, was tested in combination with carboplatin in patients with recurrent platinum sensitive OC. Cetuximab showed modest activity in these patients [230]. Panitumumab, a human monoclonal antibody specific to EGFR, in combination with carboplatin did not improve efficacy or progression free survival in platinum sensitive EOC patients [231].

## 8. Conclusions and Future Perspectives

Several studies in the last decade have associated increased inflammation and inflammatory mediators with increased EOC risk and reduced survival in EOC patients. We have presented published evidence suggesting that inflammation and inflammatory mediators promote ovarian tumorigenesis. However the mechanisms by which the process of inflammation culminates in ovarian tumor initiation need to be further understood. Such links have been established in colon and pancreatic cancer. Understanding these mechanisms is important for developing ways to target inflammatory mediators and reduce OC risk. Furthermore, epidemiological studies of NSAIDs and early clinical trials targeting IL-6 and TNF-α have shown significant promise, thus suggesting that targeting inflammatory mediators as treatment for OC warrants future research.

## Figures and Tables

**Table 1:** Role of inflammatory mediators in different stages of tumor progression.

Inflammatory Mediators	Secreting Cell Type	Stages in Tumor Progression			
		Initiation and Progression	Angiogenesis	Metastasis	Chemoresistance
TNF-α ligands, TNFRI, TNFRII	OC cells, infiltrating monocytes, macrophages	↑ autocrine production of TNF-α and IL-6, M-CSF, CXCL2, CCL2 [93,94] and AIDS mRNA level [90]	↑ VEGF, VEGF↑ CXCL12 and promotes angiogenesis [139,140,141]	↑ TGF-α secretion by stromal fibroblasts which promote peritoneal metastasis [148] Enhances migration of OC cells towards CXCL12 [149,150]	
IL-6	Ovarian epithelial cells, OC cells, M2 macrophages, mesothelial cells, TAMS, ascites	↑Proliferation by promoting G1 to S transition and MAPK-ERK- Akt activation and STAT3 activation [101,102] ↓IL-2, resulting in immune suppression [103]	Induces STAT3 and MAPK phosphorylation which enhances migration of endothelial cells [143] sIL-6R-IL-6 facilitates angiogenesis in cells lacking IL-6 receptor [144]	Stimulates production of MMPs in OCs which ↑ invasion and migration [101,104] ↑ IL-6 in ascites enhances invasion via JAK-STAT signaling [151]	↓ Caspase- 3 cleavage and makes OC cells resistant to cisplatin and paclitaxel [152] ↑Expression of MDR1, GSTpi, Bcl-2, Bcl-xL, and XIAP [152]
IL-8	Pre-ovulatory follicles, OC cells, ascites	↑ Proliferation by ↑ cyclin B1 and cyclin D1 via pAkt [110]	↑ Expression of VEGF, MMP-2, MMP-9 promoting angiogenesis [110]	Activates TAK1/ NF-κB via CXCR2 [153]	Blocks TRAIL induced apoptosis to promote resistance [154]
LPA	Follicular fluid, corpus luteum, OC cells, ascites	↑ IL-6 and IL-8 via NF-κB and AP-1 [113,114,122] ↑COX-2 AND MMP2 [115,120,121] ↑ phosphorylation of Akt and ERK resulting in increased cell cycle [123,124]	↑ Expression of VEGF via Myc and Sp-1 [155]	↑ urokinase, which results in degradation of basemembrane protein to promote metastasis [156,157]	
Prostaglandins, COX-1 and COX-2	Ovary, FT, uterus, MDSCs	↑ CXCR4 and SDF1 in MDSCs resulting in immune suppression [128]	↑ Bcl-2 and blood vessel formation [158,159]		↑ Bcl-2, thus inhibiting apoptosis in lung, colon, breast and prostate cancers [158,159]
TGF-β and EGF	OC cells, CAFs			TGF-β ↑ VCAN, which activates NF-κB and ↑MM-9 [160]	↑ EGF protects cells from cisplatin-induced apoptosis [161]. Inhibiting TGF-β sensitizes resistant cells [162]
